# Distant Presence: Care Workers' Experiences of Digital Monitoring Systems in Dementia Care

**DOI:** 10.1111/scs.70251

**Published:** 2026-06-15

**Authors:** Clara Iversen, David Redmalm, Marcus Persson, Elin Thunman

**Affiliations:** ^1^ Department of Social Work Uppsala University Uppsala Sweden; ^2^ School of Health, Care and Social Welfare, Division of Sociology Mälardalen University Västerås Sweden; ^3^ Department of Behavioural Sciences and Learning, Division of Education and Sociology Linköping University Linköping Sweden; ^4^ Department of Sociology Uppsala University Uppsala Sweden

**Keywords:** care workers, dementia care, digital technology, ethnography, monitoring, presence

## Abstract

**Aims and Objectives:**

To add nuance to when and how remote technologies should be used to support dementia care, we examine care workers' experiences of two phone‐based digital systems used for monitoring and planning in daily care.

**Methodological Design and Justification:**

Short‐term ethnography at three care homes, 49 qualitative interviews, and five video‐recorded situations provided data for a detailed account of care workers' experiences of the digital systems. Latour's concept of “presence in absence” was used to theorise the emergent theme of distant presence.

**Ethical Issues and Approval:**

Participants were given information about the project, asked to provide written consent, and could stop their participation at any time. The study was approved by the Swedish Ethical Review Authority.

**Research Methods:**

Ethnographic notes and interview transcripts were analysed using reflexive thematic analysis to identify recurrent and meaningful themes. Video recordings were transcribed and analysed using multimodal conversation analysis to illustrate how the technology affected the progression of interaction.

**Results:**

The notion of distant presence highlights how digital technology can support care work by enabling presence at a distance: Reducing disturbances and increasing efficiency. At the same time, it captures how digital tools may undermine ethical care when alarms introduce the distant into the present—competing with patients for attention—or when plans fail to adjust to the specific conditions of dementia care.

**Study Limitations:**

All participants had worked in dementia care for several years, so the experiences of new care workers were not studied.

**Conclusions:**

The findings provide insights into the complexities of how digital tools can both support and obstruct care: By introducing distant presence, digital tools can simultaneously protect patients' integrity and create a false sense of safety.

## 1 Introduction

In dementia care in the Nordic countries, digital technology is today applied to enhance security, offer stimulation for patients, and reduce staff's routine tasks Mattsson [1]. For instance, sensors are used to monitor patients, robot animals are used to support social interaction, and digital planning tools are implemented to reduce the time away from patients (Persson et al. [2]). A key question in care research has been how relationship‐based care cultures are affected, transformed, or challenged as these technologies are introduced (e.g., Ramvi et al. [3]; [4]).

In this study, we focus on this question in relation to two digital systems for planning and monitoring, asking: *How do care workers experience the digital tools in their daily work?* Previous research on digital technology and dementia has primarily addressed the experiences of patients and their relatives in care at home, reflecting a focus on support to patients to ‘age in place’ (Persson et al. [2] Ditton et al. [5]). To complement current research with nuanced knowledge about when and how remote technologies should be used to support dementia care, this study provides a detailed account of paid care workers' interactions with patients and digital systems.

## 2 Previous Research

One of the most common areas of technological development in dementia care has been tools connected to safety and monitoring [6]. Research in this area has shown that potential benefits include less disturbance of patients at night, reduced time spent on surveillance, and reduced anxiety among staff and relatives about potential adverse events [7].

In studies of users' experiences of these tools, previous research has often focused on home care and the perspectives of relatives and patients Ditton et al. [5]. This research highlights ethical dilemmas between privacy and security ([8]; Sanchez et al. [9]). For example, an interview study shows that persons with dementia were seldom aware of the technology, while their relatives viewed it as making life easier and safer (Malmgren Fänge et al. [10]). In particular, relatives emphasised the positive aspect of being able to monitor their relatives from a distance, which gave them a sense of control (see also [11]). Patients voiced fears of losing human contact but acceptance of technological systems if they strengthened their sense of security [12]. In another study, Olsson et al. [13] showed that relatives experienced monitoring devices as helping people with dementia to maintain their independence.

However, relatives also reported a number of problems, such as the unreliability of sensors, a lack of fit between users' needs and sensor functions, difficulties in obtaining consent from their relatives, and alarm fatigue stemming from the sensors' poor ability to distinguish between risky and safe situations [13, 14, 15]. Although based on accounts from relatives and patients, these studies highlight the importance of examining the experiences of technology users.

Care workers' experiences of digital technology have been studied in other care contexts, such as mental health [16] and hospitals [17]. Studies show that the relationship with the patients was a crucial element for technology to be perceived positively [16] and that the introduction of new technology requires adjustments in routines, tasks, and responsibility [17].

A small but growing body of research has investigated how technology figures in care worker–patient interactions, drawing on interviews, ethnography, and video‐based studies [18, 19, 20]. For instance, studies of pet robots in dementia care (e.g., Iversen et al. [21]; [4]) show that strict guidelines about transparency (SMER [22]) may clash with patient‐centred care in actual practice. The current study thus contributes to the understudied field of paid care workers' experiences of technology in dementia care; in particular, the actual use of digital monitoring and planning tools in daily work.

## 3 Theoretical Approach

Science and technology studies have proven useful for understanding the role of technology in care work with older people (e.g., [4, 23, 24]). In this article, we draw on Latour's [25] argument that science is connected to researchers' ability to bring back traces from their studies, allowing an inexperienced actor to “be familiar with things, people and events, which are distant” ([25], 220). Scientific devices, including immaterial devices such as statistics, provide means for making phenomena: (a) mobile, so that they can be brought back from the field, (b) stable, so that they can be moved without being altered, and (c) combinable, so that they can be accumulated, aggregated, and mixed ([25], 232). In this way, scientific knowledge enables presence in absence—the actual person or event studied does not need to be present for the scientist to draw conclusions. However, the challenge is that the resulting information requires that the element which it represents be stable ([25], 254). Thus, scientific knowledge is valuable only as long as its objects remain recognisable within the scientific regime.

While Latour's concept of presence in absence has not previously been applied to understanding technology in care, it is evident that monitoring technologies enable presence from a distance (see Malmgren Fänge et al. [10]). Care workers can gain knowledge about events that are physically distant—e.g., noticing that someone in a room across the building is moving in ways that may involve a risk of falling. However, just as scientists must rely on the stability of elements, this technology rests on the predictability of risky and safebehaviours. Otherwise, the knowledge generated will not be ‘combinable’—it will be difficult for caregivers to share and learn from if it is not grounded in shared understandings of potential risks and best practices. Thus, we employ the concept of “distant presence” to highlight the possibilities and limitations of the digital tools under study.

In particular, we are interested in what happens when digital technology is put to use in daily care. Scholars have noted that a key factor in whether technology is experienced as useful is its ability to adapt to patients' habits. Many systems rely on behaviour modelling techniques to identify warning signs, making it important that they do not lock users into routines that no longer suit them [26]. Berridge [27] warns that the expectation of strict routines, coupled with the absence of options for older people to exercise control over the system, may cause difficulties for some users. The older adults she interviewed about home‐based sensors expressed hyper‐vigilance to avoid unnecessary alerts that could distract family members. Consequently, the system played an institutionalising role by policing behaviours that might trigger alarms, such as staying in the bathroom or taking an afternoon nap for ‘too long’ [27]. In Latour's terms, we can talk about a lack of fit between presence and absence. In the analysis, we will show how the concept of distant presence enables us to, on the one hand, understand desired efficiency and avoidance of disturbances as enabling presence from a distance, and on the other, unwanted presence of inflexible alarms that constantly call attention to the distant.

## 4 Methods

### 4.1 Study Design

The study employed a short‐term ethnographic design [28], including observations at three care homes for periods of two to eight days, 49 interviews with care workers (including care managers) at seven care facilities, and video recordings of five social situations in one care home. The combination of interviews and ethnographic observations allows us to study people's accounts as well as their sense‐making in natural contexts (see [29]). Our video recordings further enriched the approach by enabling detailed examination of relevant interactions [30].

In the current article, we focus on two digital tools: The first is a monitoring system (Sensio) that transmits data to the care worker's phone without the user's action or awareness. Data include falls, location, activity, and gait speed. The second system is a planning tool (Epsilon), linked to another phone, which lists care workers' daily schedules and tasks for different patients. While the systems have quite different functions, the care workers talked about them in similar ways—probably because they were both accessed via phone applications, which conflict with previous guidelines discouraging the use of phones in the presence of patients. In addition, both systems involve monitoring—Sensio of patients' behaviour in relation to calculated risk and Epsilon of care workers' time with patients in relation to a calculated ideal. They, therefore, both illustrate presence in absence.

### 4.2 Sample, Data Collection, and Analysis

We contacted care homes based on recommendations from a reference group tied to the project, which included representatives from Swedish organisations working with care and technology. We first presented the study to managers and care workers, who then provided written consent to participate. To capture care workers' experiences of applying technology in the daily routines of care work, we included participants who had worked in dementia care for at least two years. Therefore, we excluded the experiences of using technology while being new to dementia care. For ethnographic observations and video recordings involving patients, the patients were given time to consider participation after receiving letters about the study. Care workers asked patients who they assessed would be able to actively consent to participation and provided them with written information. While relying on care workers' assessment may have excluded patients who wanted to participate in the study, our relatively short‐term ethnography did not allow us to get to know the patients well enough to make this judgment ourselves. During our fieldwork, care workers introduced us to patients who had provided written consent to be filmed, and we requested permission again before filming after showing our video equipment. The participating patients had mild to moderate dementia.

The interviews were transcribed with the university's secure AI transcription tool, and transcripts were then checked manually. We used thematic analysis to identify recurrent and meaningful themes [31]. In an initial close reading of interview transcripts and ethnographic notes, Iversen found that distance and presence appeared as central themes; they were both frequent and important for care workers' experiences. In the next step, the different authors critically examined data, looking for deviant cases, which would contradict or develop the analysis. The theme recurred in interviews and ethnographic data and over discussions of different technological tools, which led us to conclude that we had reached data saturation (see Saunders et al. [22, 32]).

Having identified this theme, we applied multimodal conversation analysis to the video data to illustrate our findings. Multimodal conversation analysis is based on conversation analysis, an empirically driven and microanalytic method used to analyse how people, turn by turn, make themselves understandable in social situations (e.g., [30]). Beyond verbal conduct, multimodal conversation analysis incorporates the material aspects of interaction by drawing on video recordings and transcripts that show how embodied practices are timed in relation to verbal practices—e.g., how a gaze can indicate that an utterance is directed at a particular person. In this article, multimodal analysis allows us to illustrate step by step how a digital alarm can influence the progression of an interaction. The transcripts include a simplified multimodal transcription with comments on embodied conduct and line drawings based on frame grabs from the videos (see Appendix 1 for transcription conventions).

## 5 Ethical Considerations

The study was approved by the Swedish Ethical Review Authority [DNR 2023‐05‐17]. A key ethical dilemma was to ensure that patients who wished to participate in the research were not excluded based on others' judgements, while also preventing participation by those who did not fully understand the content of the research and could therefore not provide informed consent. Participants could stop the filming at any time and request that recordings be deleted (i.e., process consent). In addition, we took care to present examples in ways that prevented care workers and care managers from being recognisable to one another or to others, focusing on themes rather than individuals.

In the following section, we present the findings in three sub‐sections. The transcripts include only English translations to facilitate reading. All names have been replaced with pseudonyms.

## 6 Care Workers' Experiences of Digital Monitoring and Planning Systems

The analysis shows that the care workers perceived digital technology—both monitoring and planning tools—as creating presence at a distance and distance despite presence. In what follows, we demonstrate how distant presence can reduce disturbances and promote efficiency, but also introduce digital features that compete with patients for care workers' attention and care ideals.

### 6.1 Reducing Disturbances and Promoting Efficiency

The digital systems are described in positive terms as reducing tasks that interfere with a caring relationship and by promoting efficiency. For instance, a care worker says that the digital alarm has enabled the staff to better respect dementia patients' integrity by limiting their manual surveillance:

Having digital supervision means that when they go to the toilet, some can manage and then we don't need to run in to their room […] so they can manage in peace and quiet (D1).

The digital technology is presented as liberating staff from unnecessary check‐ins, preserving “peace and quiet.” Even if patients are monitored to the same extent—or even more intensively—the task is now digitalised, and the relationship between care workers and patients is freed from unwanted disturbances. Instead, the interactions are characterised by respect and trust. Another care worker (VÖ1) says:

The alarms are great, especially if you're working alone. Because you can go in and check the camera instead of going in and disturbing them. If they're not on their way up, then you go in. If you want to check something… […] Some just get up to go to the toilet, and instead of going in and making them anxious, you can wait two or three minutes and, “okay, now he or she is back”.

In addition to allowing staff to check in without causing patients worry, which may happen when dementia patients receive unexpected visits, the digital alarm is also seen as promoting efficiency when working alone. Care workers thus approach the use of digital alarms as a way to provide ethical care from a distance.

Related to the digital planning tool, the care workers also describe efficiency as a key aspect of being present from a distance. A care worker (MÄ11) reports how time is freed from physical transportation:

You can immediately see what needs to be done: “It's those and those who need help.” Before, you had to read lists or sign by hand. And you had to go away to help someone, or give someone a pill, and then walk all the way back.

The care worker notes the benefits of immediate access to information, not needing to search for information or physically move between different patients. This is understood as enhancing ethical care by reducing the time spent on administrative tasks in favour of patient‐centred care. In addition, the planning tool is seen as increasing safety by providing more reliable information than human memory. A care worker expresses relief in being able to rely on a scheduled “loop,” visible via an app in a mobile phone: “I think it's good because you don't forget any medication” (D2). Remembering practical tasks, such as giving medication, is part of dementia care. The digital technology thus removes possible disturbances related to uncertainty by offering knowledge, memory, and monitoring from a distance.

Some interviewees expressed concern about overreliance on technology and uncertainty about who is actually in control, but such problems were generally seen as manageable with training. During the fieldwork, we noted that care workers had developed what we could call ‘alarm competence’ to handle different kinds of alarms. Here, an alarm categorised as “false” goes off as the researcher (R) is present (VÄ3) and the care worker (CW) turns it off without further checking:

CW: It's an alarm, but no one lives there. I'll just turn it off.

R: Oh, a false alarm?

CW: Because there's no one in the room, I know that. There was a girl cleaning in there; maybe it was her. Sometimes it happens. But no one lives there now.

The care worker's knowledge about the care facility, as well as how the alarm works, allows her to dismiss it. With this approach, care workers can use digital tools to gather information from a distance while they see themselves as responsible for assessing this information. This approach was also visible in the initial examples in this section, where the care workers describe being able to calmly evaluate monitoring information without involving patients.

### 6.2 Competing for Attention

While care workers talked about the benefits of not disturbing patients, they often described being distracted themselves. The core of this problem is tied to attention and the physical impossibility of remaining fully present while constantly being flooded with signals:

And then it beeps. I know that my customers [patients] are here. […] But one still has to look [at the phone], so it's constantly like this because it beeps everywhere. It is stressful. Really stressful (L2).

We can see here how the benefits of technology—making it possible to monitor what is distant—disturb care in the present through this very function: Although the care workers are in the presence of patients, they must look at their phones because the alarm demands their attention. We could sometimes observe situations similar to the one described in the quote above. In the following example, from a videotaped visit in a patient's apartment, we examine moment by moment how the digital alarm competes with the patient for the care worker's attention.

**FIGURE 1:**
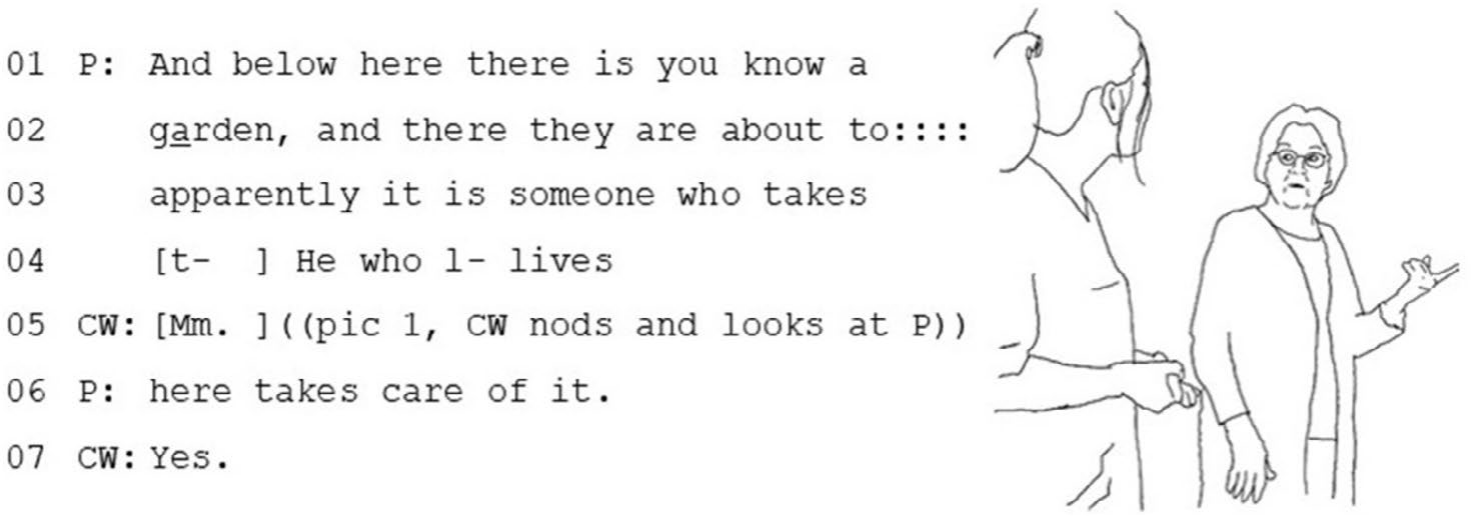
Garden story.

We enter the interaction (Figure 1) as the patient (P) tells the care worker (CW) about a garden outside her window, organised by a neighbour. The care worker participates actively in the conversation while the researcher (R) remains in the background, holding the camera:

The patient's gaze is directed at the care worker, indicating that the story is addressed to her (lines 03–05, pic 1). We can see the care worker reciprocating this attention by participating as the main recipient of the patient's story. She uses both verbal and embodied resources, looking at her, nodding, and encouraging her to go on with “Mm” (line 05) and “Yes” (line 07).

**FIGURE 2:**
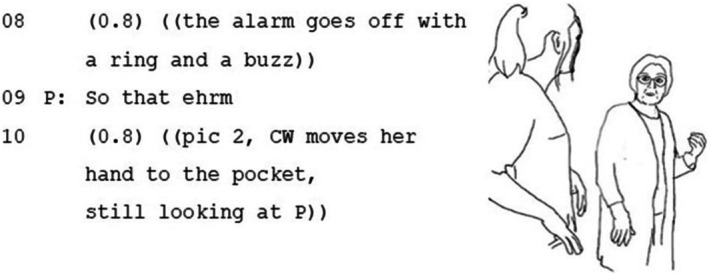
The alarm goes off.

**FIGURE 3:**
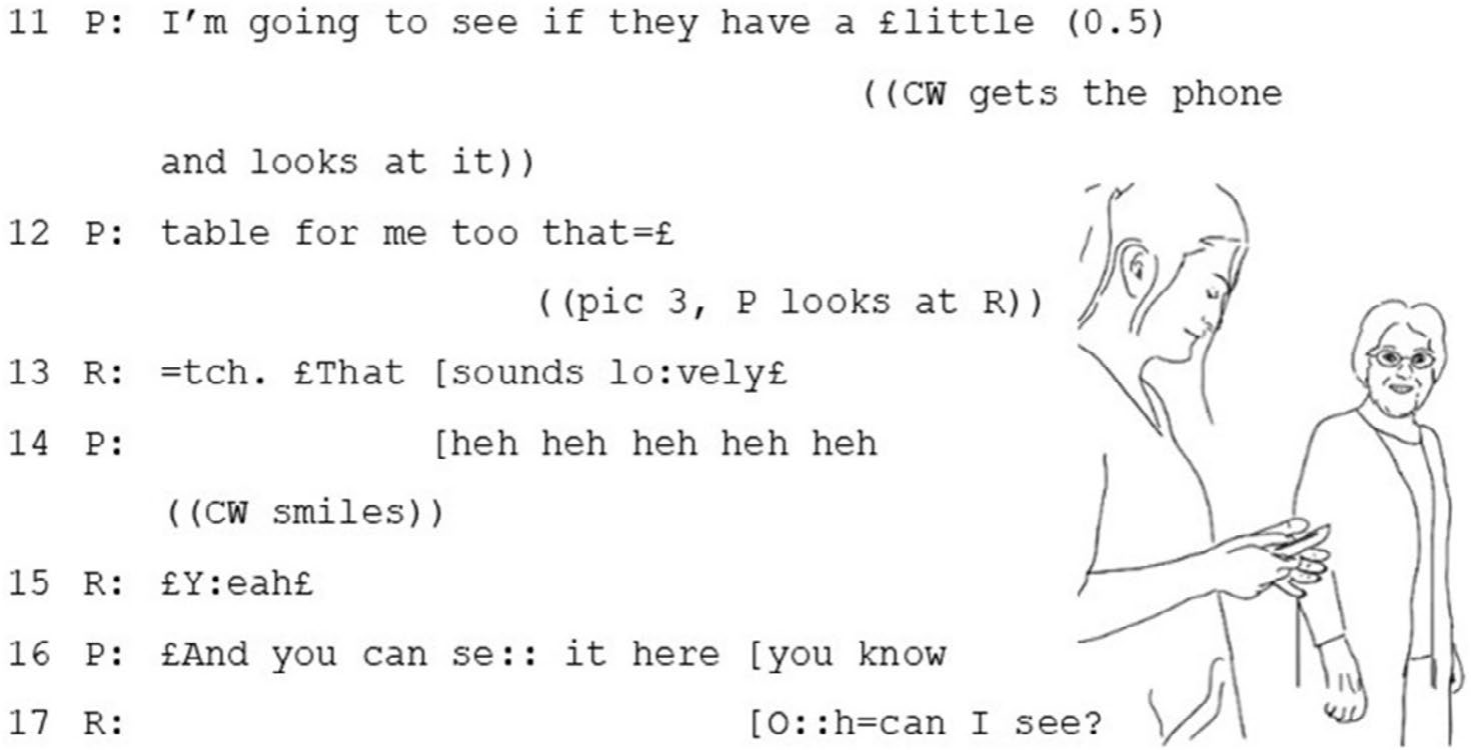
The researcher participates.

In the next excerpt (Figure 2), we follow how the interaction changes as the alarm goes off on the care worker's phone:

We can note that the patient stops her story about the garden at the exact moment the alarm is first heard (line 08). When she begins her next turn, her speech is prolonged by an “ehrm” (line 09), indicating difficulty continuing. The care worker maintains her gaze on the patient while simultaneously moving her hand to her pocket where the phone is located (line 10, pic 2). This shows how the alarm negatively affects the flow of the conversation. At the same time, we see the care worker's effort to keep her attention on the patient. The patient resumes her story (Figure 3), saying that she will ask the neighbour about getting a table of her own (lines 11–12) while the care worker picks up the phone and looks at it:

The patient responds to the care worker's lack of attention by shifting her gaze to the researcher (line 12), who accepts this more active role by providing a positive assessment (“That sounds lovely,” line 13). Thus, the patient and the researcher become the main participants in the interaction (lines 16–17). The care worker continues to participate in the conversation by smiling as the patient laughs, but she looks down at the phone (line 14). Eleven seconds after this transcript ends, she puts her phone down and reengages with the patient.

This example helps us see how the digital alarm affects the interaction between a patient and a care worker by interrupting the conversation and demanding the care worker's attention. While this is a single‐case analysis, potential threats to the caring relationship are evident in how the patient seeks a response from another present party, the researcher. In addition, we observe the dividing effects of the alarm in the care worker's efforts to remain attentive to the patient while checking her phone.

That care workers sometimes delay responding to alarms—and sometimes do not respond at all—can, as we saw in the previous section, be understood as alarm competence. However, in situations like this, we can also see how delayed or absent responses to alarms may be connected to alarm fatigue and the clash between the here and now and the distant (VÄ4). This poses a threat to care in practice.

### 6.3 Responsiveness Vs. Plans: Care Ideals at Risk

The care workers describe the challenge of remaining fully present in the moment and the rigid schedule provided by the digital planning tool as a risk to care ideals, emphasising a calm and safe environment where they can promote patients' communicative skills.

In the interviews, care workers talk about the physical redirection of attention as a moral problem. Several interviewees note that “it looks bad” for both patients and visiting relatives (L2; Q1). What might seem like a superficial moral problem (how it looks) can become a real moral problem related to a lack of attention toward patients. A care manager reflects on how the constant use of mobile phones may lead patients to feel ignored:

Since it is a phone, it's so tied to other things, I don't know actually how it might affect the customer [patient], but some customers have said, “they [care workers] just sit over there with their phones” (Q1).

The collision between patients' needs and digital technology can also be understood in relation to planning in the past vs. responding to needs in the present:

With dementia, it's a bit difficult because you can't follow the routine. After all, with dementia, things happen—she will wake up earlier, the other will wake up later, and she wants breakfast. (D1)

We can understand this as digital technology creating a norm around the patient—a datafication of needs. Some care workers were also concerned that the time spent on making digital systems work was time poorly spent:

It takes … maybe a few seconds or a minute when you log in or out. This time can be spent with our residents [patients] who are here. We can … sit and talk with them during this time (D2).

We can note here the difference between the patients who are “here” and the distant reality represented by the phone—a task external to the immediate caring relationship. This contrasts with the first analytic section, where a care worker talked about immediate access to the distant as providing *more* patient time.

Thus, in the latter sections, we have seen the negative side of technology's ability to create presence from a distance, manifesting as the unwanted presence of phones, cumbersome login procedures, noise, and rigid time management, which collide with ideals of attention, flexibility, presence, and calmness—ideals that the interviewees say are necessary in dignified care for dementia patients (also see [33]).

## 7 Discussion

The concept of distant presence allows us to shed light on both intended and unintended consequences of the digital systems we studied. Below, we discuss the implications of these findings in terms of a conflict between transparency and *experienced* nonintrusiveness, and of alarms as a work environment problem.

### 7.1 The Lack of Transparency of Nonintrusive Technology

Similar to previous research findings, we found accounts of digital technology as creating efficiency and a sense of safety without requiring workers to constantly impose their presence on patients (see [7, 12]). Drawing on Latour [25], we understand this as enabling a form of presence—a watchful and caring eye—from a distance. These findings align with benefits that relatives have described in previous studies (see [11, 15]). In the current study, dignity‐preserving practices at a distance were related to specific situations: Toilet visits and unexpected check‐ups. In such situations, digital monitoring may be useful because, unlike a human, it captures only safety‐related information and, in addition, because the patients are unaware that they are being monitored.

However, this makes visible a gap between the *experience* of non‐intrusiveness and actual surveillance, linked to conflicting ethical ideals of security and privacy ([8]; Sanchez et al. [9]). Previous research shows that patients with dementia may not be aware that they are monitored [15], and our findings show care workers treating this as a benefit, as it does not disturb patients. By contrast, transparency is central in medical guidelines on dementia care (e.g., SMER [22]). In relation to pet robots, research suggests that such guidelines may not resonate with patient‐centred care and that care workers often prefer white lies to reminding patients that their pets are ‘not real’ (Iversen et al. [21]; [19]). Our findings show a similar priority of creating a safe and calm environment over transparency, and this mismatch with guidelines stresses the need to continue exploring patients', relatives', and care workers' different perspectives on transparency vs. experienced non‐intrusiveness in dementia care.

### 7.2 Constant Alarms as a Work Environment Problem

In relation to problems arising from a mismatch between expectations of regularity embedded in digital tools and older persons' need for flexibility, this study finds similar patterns as previous studies [26, 27]. While previous research has focused on patients' experiences, we shed light on this as a work environment problem. In particular, our findings show how the expectation of controlling things from a distance may collide with care workers' ideals of ethical care in the present—both temporally and spatially. The constant beeping, both from the monitoring system and from the planning tool, introduced distance—from other patients' alarms and from a predetermined schedule—into the presence of face‐to‐face interactions and current needs. The fact that both systems we studied were present in the form of mobile applications meant that care workers had to constantly look at their phones—a practice that ran counter to the care homes' previous mobile phone guidelines. This distracted care workers and also made them think that patients felt ignored. Thus, introducing distance into present situations was another negative side of digital monitoring and planning tools.

## 8 Distant Presence and Alarm Competence

With Latour's notion of presence in absence, the study captures the complexity of digital monitoring and planning in dementia care. Translated to this study, absence in the form of distance requires not only mobile information and stable communication channels, but also that information is ‘combinable’—so that caregivers can share and learn from it—and that it does not replace everyday exchanges between care workers and patients.

While we noted that what we term ‘alarm competence’—approaching technology based on knowledge and experience—was an important element in positive accounts of the technology, it is important that this does not turn into ‘alarm fatigue,’ in which information external to the immediate situation is dismissed (see [14, 15]). Previous research has highlighted that the introduction of new technology requires adjustments in routines, tasks, and responsibilities (Åsgard and Aasum [34]). Similarly, this study points to the need to allocate resources in the introduction of digital systems, which can support the development of alarm competence. Furthermore, the work environment problem of constant alarms highlights that care workers need time off from the alarms to be able to focus their attention on patients, being fully present.

In this way, distant presence is not only a theoretical concept for understanding the tensions of digitalisation in care but also a practical tool for reflecting on when and how remote technologies should be used to support both care workers and patients.

## 9 Limitations

A strength of the study is the identification of similar themes across different care homes, distinct sources of data, and care workers with diverse positions and educational backgrounds. However, our focus on care workers who had worked for at least two years means that we have not accounted for the experiences of short‐term or new staff. This is a substantial share of staff in Swedish dementia care, and further research is needed to examine their experiences, in particular in relation to the development of ‘alarm competence’. Further research is also needed on patients' and relatives' experiences of staff's use of mobile phones.

## 10 Conclusion

Technology is rapidly being introduced in various domains of dementia care, and it will undoubtedly shape our understanding of ageing and care. With the aim of examining how care workers experience digital monitoring and planning tools in their daily work, we have shown how presence from a distance can support care work by reducing unwanted disturbances and promoting efficiency. However, we also show how the introduction of distance in present situations can risk undermining care ideals when digital tools compete with patients for attention and structure work in ways that are not attuned to dementia patients' needs.

The findings illuminate what happens when digital tools are introduced in dementia care; in particular, the study offers nuances to our understanding of how digital resources can both support and obstruct care. It is crucial that digital systems do not disturb patients, but the lack of disturbances may come at the expense of patients' awareness of being monitored. In addition, digital systems risk decreasing the physical time spent with patients, but sometimes, using these systems to quickly gather information about patients or reducing the time spent going from patient to patient may enable ethical care from a distance in an otherwise present relationship. Finding a balance between the problems and affordances of digital resources is key to ensuring that core care values are not lost in the search for efficiency. Future studies, therefore, need to examine issues of non‐intrusiveness and informed consent from patient perspectives, and further study the impact of alarms on care workers' work environment.

## Author Contributions

CrediT: Clara Iversen:Conceptualisation, Formal analysis, Methodology, Writing – original draft, Funding acquisition, Investigation; David Redmalm: Formal analysis, Investigation, Methodology, Writing – review and editing; Funding acquisition; Marcus Persson: Funding acquisition, Investigation, project administration, Writing – review and editing; Elin Thunman: Funding acquisition, Methodology, Writing – review and editing.

## Funding

This work was supported by AFA Försäkring (220257).

## Ethics Statement

The study was approved by the Swedish Ethical Review Authority [DNR 2023‐05‐17].

## Consent

All patients included in the study have provided written consent.

## Conflicts of Interest

The authors declare no conflicts of interest.

## Data Availability

The data that support the findings of this study are available on request from the corresponding author. The data are not publicly available due to privacy or ethical restrictions.

## Appendix 1

Transcription Conventions. Extract from Gail Jefferson's (2004, 24–31) transcription symbols. Jefferson G (2004) Glossary of transcript symbols with an introduction. In Lerner, GH (ed.) *Conversation Analysis: Studies from the first Generation*. Amsterdam: John Benjamins Publishing, 13–31.

**Table:**

[	Onset of overlap
]	The point at which two overlapping utterances end
=	Latching, no break or gap
(0.0)	Elapsed time by tenth of seconds
(.)	Micro pause
word	Stress via pitch and/or amplitude
:	Prolongation of the immediate prior sound
↑↓	Shifts into especially high or low pitch
,?.	Punctuation marks are used to indicate the usual intonation
WORD	Louder sounds
°word°	Softer or quieter sound
wo—	Cut off
> word<	The bracketed sounds are sped up
< word>	The bracketed material is slowed down
.hhh	Inbreath
hhh.	Outbreath
wo(hh)rd	Laughter particles in word
£word£	Smiling voice
((word))	The transcriber's comments about embodied actions, placed at the onset of the action
(word)	The transcriber's guess
Pic #	Picture – line drawing made from a video screenshot.

## References

[1] T. Mattsson , “The Digitalization of Elderly Care in Sweden and Europe,” Welfare Issue Today 142, no. 1 (2025): 10–11, https://www.welfare.seoul.kr/web/contents/archive1‐1.do.

[2] M. Persson , D. Redmalm , and C. Iversen , “Caregivers’ Use of Robots and Their Effect on Work Environment – a Scoping Review,” Journal of Technology in Human Services 40, no. 3 (2022): 251–277, 10.1080/15228835.2021.2000554.

[3] E. Ramvi , I. Hellstrand , I. B. Jensen , B. H. Gripsrud , and B. Gjerstad , “Ethics of Care in Technology‐Mediated Healthcare Practices: A Scoping Review,” Scandinavian Journal of Caring Sciences 37, no. 4 (2023): 1123–1135, 10.1111/scs.13186. 37272481

[4] D. Redmalm , C. Iversen , and M. Persson , “Can Robots Lie?: A Posthumanist Approach to Robotic Animals and Deceptive Practices in Dementia Care,” Journal of Aging Studies 71 (2024): 101272, 10.1016/j.jaging.2024.101272. 39608910

[5] A. Ditton , H. Alodan , C. Fox , S. Evans , and J. Cross , “Exploring the Effectiveness and Experiences of People Living With Dementia Interacting With Digital Interventions: A Mixed Methods Systematic Review,” Dementia 24, no. 3 (2025): 506–551, 10.1177/14713012241302371. 39604136 PMC11915779

[6] K. Lorenz , P. P. Freddolino , A. Comas‐Herrera , M. Knapp , and J. Damant , “Technology‐Based Tools and Services for People With Dementia and Carers: Mapping Technology Onto the Dementia Care Pathway,” Dementia 18, no. 2 (2019): 725–741, 10.1177/1471301217691617. 28178858

[7] A. Hall , C. B. Wilson , E. Stanmore , and C. Todd , “Moving Beyond ‘Safety’ Versus ‘Autonomy’: A Qualitative Exploration of the Ethics of Using Monitoring Technologies in Long‐Term Dementia Care,” BMC Geriatrics 19 (2019): 6–19, 10.1186/s12877-019-1155-6. 31126240 PMC6534927

[8] A. R. Niemeijer , B. J. N. Frederiks , I. I. Riphagen , J. Legemaate , J. Eefsting , and C. M. Hertogh , “Ethical and Practical Concerns of Surveillance Technologies in Residential Care for People With Dementia or Intellectual Disabilities: An Overview of the Literature,” International Psychogeriatrics 4 (2010): 1–14, 10.1017/S1041610210000037. 20199699

[9] V. G. Sanchez , I. Taylor , and P. C. Bing‐Johnson , “Ethics of Smart House Welfare Technology for Older Adults: A Systematic Literature Review,” Informatics for Health and Social Care 47, no. 1 (2017): 10–37, 10.1017/S0266462317000964. 29151393

[10] A. Malmgren Fänge , G. Carlsson , C. Chiatti , and C. Lethin , “Using Sensor‐Based Technology for Safety and Independence – the Experiences of People with Dementia and Their Families,” Scandinavian Journal of Caring Sciences 34, no. 3 (2020): 648–657, 10.1111/scs.12766. 31614031

[11] M. K. Gullslett , E. R. Nilsen , and J. Dugstad , “Relatives's Experiences With and Attitudes Towards Digital Monitoring Technology for Ageing People With Dementia in Residential Care Facilities. A Qualitative Study Based on the Voices of Relatives and Care Providers,” Scandinavian Journal of Caring Sciences 36, no. 4 (2022): 1094–1103, 10.1111/scs.13009. 34121217

[12] M. Sallinen , O. Hentonen , and S. Teeri , “Ethical Dilemmas Related to the Use of Safety Technology in Service House Environments,” Scandinavian Journal of Caring Sciences 34, no. 1 (2020): 199–205, 10.1111/scs.12721. 31250937

[13] A. Olsson , M. Engström , K. Skovdahl , and C. Lampic , “My, Your and Our Needs for Safety and Security: Relatives' Reflections on Using Information and Communication Technology in Dementia Care,” Scandinavian Journal of Caring Sciences 26, no. 1 (2012): 104–112, 10.1111/j.1471-6712.2011.00916.x. 21843198

[14] M. Cvach , “Monitor alarm fatigue: An integrative review,” Biomedical Instrumentation & Technology 46, no. 4 (2012): 268–277, 10.2345/0899-8205-46.4.268. 22839984

[15] A. Malmgren Fänge , G. Carlsson , C. Chiatti , and C. Lethin , “Using Sensor‐Based Technology for Safety and Independence – The Experiences of People With Dementia and Their Families,” Scandinavian Journal of Caring Sciences 34, no. 3 (2020): 648–657, 10.1111/scs.12766. 31614031

[16] F. Forma , K. Chiu , J. Shafrin , D. H. Boskovic , and S. P. Veeranki , “Are Caregivers Ready for Digital? Caregiver Preferences for Health Technology Tools to Monitor Medication Adherence Among Patients With Serious Mental Illness,” DIGITAL HEALTH 8 (2022): 20552076221084472, 10.1177/20552076221084472. 35295765 PMC8918958

[17] F. C. Åsgard and L. Aasen , “Digital Home Monitoring—Responsibilities and Task Distribution: A Qualitative Study of Health Professionals in Hospitals,” Scandinavian Journal of Caring Sciences 39, no. 4 (2025): e70152, 10.1111/scs.70152. 41193415

[18] M. Persson , C. Iversen , and D. Redmalm , “Making Robots Matter in Dementia Care: Conceptualising the Triadic Interaction Between Caregiver, Resident and Robot Animal,” Sociology of Health & Illness 46, no. 6 (2024): 1192–1211, 10.1111/1467-9566.13786. 38733615

[19] M. Persson , L. Ferm , D. Redmalm , and C. Iversen , “Working With Robotic Animals in Dementia Care: The Significance of Caregivers' Competences. Nordic Journal of Working Life,” Studies 13, no. 3 (2023): 49–69, 10.18291/njwls.136521.

[20] J. Rondon‐Sulbaran , J. Daly Lynn , B. McCormack , A. Ryan , and S. Martin , “The Transition to Technology‐Enriched Supported Accommodation (TESA) for People Living With Dementia: The Experience of Formal Carers,” Ageing and Society 40, no. 10 (2020): 2287–2308, 10.1017/S0144686X19000588.

[21] C. Iversen , M. Persson , and D. Redmalm , “Playful Framings of Social Robots in Dementia Care: Reconsidering the Principle of Transparency in Interactions With Robot Animals,” Ageing and Society 45, no. 8 (2025): 1585–1606, 10.1017/S0144686X24000539.

[22] Statens medicin‐etiska råd [SMER; The Swedish Medical Ethics Council] , Robotar och övervakning i vården av äldre – etiska aspekter. [Robots and surveillance in the care of elderly—ethical aspects] (SMER, 2014).

[23] M. Cozza , S. Bruzzone , and L. Crevani , “Materialities of Care for Older People: Caring Together/Apart in the Political Economy of Caring Apparatus,” Health Sociology Review 30, no. 3 (2021): 308–322, 10.1080/14461242.2021.1976067. 34605377

[24] A. Mol , I. Moser , and J. Pols , Care in Practice: On Tinkering in Clinics, Homes and Farms (Transcript, 2010), 10.14361/transcript.9783839414477.

[25] B. Latour , Science in Action: How to Follow Scientists and Engineers Through Society (Harvard Univ. Press, 1987).

[26] M. L. S. Lie , S. Lindsay , and K. Brittain , “Technology and Trust: Older People's Perspectives of a Home Monitoring System,” Ageing and Society 36, no. 7 (2016): 1501–1525, 10.1017/S0144686X15000501.

[27] C. Berridge , “Active Subjects of Passive Monitoring: Responses to a Passive Monitoring System in Low‐Income Independent Living,” Ageing and Society 37, no. 3 (2017): 537–560, 10.1017/S0144686X15001269. 28239211 PMC5321543

[28] H. Knoblauch , “Focused Ethnography. Forum Qualitative Sozialforschung Forum,” Qualitative Social Research 6, no. 3 (2005): 123–141, 10.17169/fqs-6.3.20.

[29] M. Hammersley and P. Atkinson , Ethnography: Principles in Practice (Routledge, 2007).

[30] J. Sidnell , Basic Conversation Analytic Methods. In the Handbook of Conversation Analysis (Pp. 77‐99) (Blackwell Publishing Ltd, 2012), 10.1002/9781118325001.ch5.

[31] V. Braun and V. Clarke , “Toward Good Practice in Thematic Analysis: Avoiding Common Problems and Be(Com)ing a Knowing Researcher,” International Journal of Transgender Health 24, no. 1 (2022): 1–6, 10.1080/26895269.2022.2129597. 36713144 PMC9879167

[32] B. Saunders , J. Sim , T. Kingstone , et al., “Saturation in Qualitative Research: Exploring Its Conceptualization and Operationalization,” Quality and Quantity 52, no. 4 (2018): 1893–1907, 10.1007/s11135-017-0574-8. 29937585 PMC5993836

[33] W. van der Geugten and A. Goossensen , “Dignifying and Undignifying Aspects of Care for People With Dementia: A Narrative Review,” Scandinavian Journal of Caring Sciences 34 (2020): 818–838, 10.1111/scs.12791. 31750569 PMC7754132

[34] F. C. Åsgard and L. Aasen , “Digital Home Monitoring—Responsibilities and Task Distribution: A Qualitative Study of Health Professionals in Hospitals,” Scandinavian Journal of Caring Sciences 39, no. 4 (2025): e70152, 10.1111/scs.70152. 41193415

